# Results of a Web-based survey of 2105 Greek migraine patients in 2020: demographics, clinical characteristics, burden and the effects of the COVID-19 pandemic on the course of migraine

**DOI:** 10.1186/s12883-022-02968-9

**Published:** 2022-11-21

**Authors:** Emmanouil V. Dermitzakis, Aikaterini Kouroudi, Andreas A. Argyriou, Konstantinos C. Spingos, Konstantinos Bilias, Michail Vikelis

**Affiliations:** 1 Department of Neurology, Euromedica General Clinic, 54645 Thessaloniki, Greece; 2 Greek Society of Migraine and Headache Patients, Athens, Greece; 3 Headache Outpatient Clinic, Neurology Department, Agios Andreas State General Hospital of Patras, Patras, Greece; 4 Corfu Headache Clinic, Corfu, Greece; 5 Headache Clinic, Mediterraneo Hospital, Glyfada, Greece

**Keywords:** Migraine, Chronic migraine, Web-based survey, Demographic and clinical characteristics, Burden, COVID-19 pandemic

## Abstract

The Greek Society of Migraine and Headache Patients (GSMHP), maintaining a strong commitment to research and information, conducted its second web-based online survey named "Migraine in Greece—2020", following its first one conducted in 2018. The 2020 study included 2,105 migraine patients who were called to answer 151 questions. The purposes of the current research were to record the demographic and clinical characteristics of migraine patients in Greece, including the severity and effects of migraine on respondents' quality of life, as well as to survey the effects of the coronavirus pandemic on the course of migraine. Our population, internet-based study provides data that will hopefully contribute to better comprehend the clinical phenotype and course of migraine during the COVID-19 pandemic.

## Introduction

Migraine is one of the most frequent primary headache disorders and constitutes one of the major causes of disability among neurological disorders [1]. Depending on its monthly frequency, migraine can be subtyped to either episodic (less than 15 days monthly with 4–8 days monthly sub-classified as low frequency and 8–14 days monthly defined as high frequency episodic) or chronic migraine (more than 15 headache days monthly, of which at least 8 are of migrainous type or respond to migraine-specific medication, for more than 3 months) [2]. The disease’s phenotype of patients with episodic migraine (EM) is generally much more benign, being characterized by shorter average duration of headache, less pain intensity, milder pain-associated autonomic symptoms and less pain-related comorbidities, compared to their counterparts with chronic migraine (CM) [3]. On the other hand, CM accounting for about 2.5% of migraineurs, carries greater disability, much reduced productivity at work/school, increased rates of pain-related comorbidities and multiple direct and indirect health costs, than EM [4], thoroughly significantly compromising the quality of life (QOL) and daily living activities of sufferers [5]. In addition, there is evidence from large population-based studies of clinical risk factors for migraine chronification, mainly including increased headache frequency; occurrence of obesity and metabolic syndrome; medication overuse headache (MOH) and psychiatric comorbidities [6].

Globally, there are associations of patients with migraine that aim to highlight the impact of the disease so as to raise public and state awareness, among other objectives, through publication of results from population-based studies conducted with the use of internet or telephone-led surveys. Nonetheless, despite previous observational studies from USA or Europe, such as the Chronic Migraine Epidemiology and Outcomes (CaMEO) Study [7], the migraine in America symptoms and treatment study (MAST) [8], the American Migraine Prevalence and Prevention (AMPP) study [9] and the Eurolight project [10], there are still issues that remain vaguely defined in relation to the demographic and epidemiological data, severity and impact of the disease in migraine sufferers, particularly nowadays during the outbreak of the COVID-19 pandemic.

Thus far, very few population-based studies on migraine are available in Greece [11]. Quite recently, the results of an observational descriptive survey, using computer-assisted telephone interviews, were released to report the prevalence and burden of selected disabling primary headache in a large sample of adult Greek population [12]. It was evident that among a total of 1197 participants, 916 (76.5%) were with migraine (813 with EM and 103 with CM), corresponding to a significant one-year prevalence; thus, supporting the view that migraine is the most common primary disabling headache disorder in Greece, because of high rates of both absenteeism and presenteeism with reduced performance; stigmatization, and significant difficulty to accomplish social as also family obligations. Despite the high burden of migraine, the majority of migraineurs participants were not under any preventative treatment [12].

On a national level, the Greek Society of Migraine and Headache Patients (GSMHP) was established in 2017. It is a non-profit association, being member of the Pain Alliance Europe and European Migraine & Headache Alliance. The main axes of its action under the support of headache specialists are (i) to inform and educate sufferers, the general public, health professionals and state officials on headaches and treatment options, (ii) to promote socialization of patients along with activation and participation in decisions concerning their health problem; (iii) to develop Research Tools and provide Education and (iv) to support claim of patients’ legitimate rights. In 2020, GSMHP formally had 1812 members and 10,000 followers in its social media accounts, including Facebook, Instagram and Twitter.

The main purposes of this current population-based survey were to record the demographic and clinical characteristics of migraine patients in Greece, including the severity and effects of migraine on patients' quality of life, as well as to survey the effects of the COVID-19 pandemic on the course of migraine. Notably, this online survey is the second that is conducted by the GSMHP, as in 2018 the results of a similar survey in a sample of 1091 patients with migraine were published in the official journal of the Hellenic Neurological Society to describe the prevalence of the disease, its societal/psychological burden and patients’ treatment preferences [13]. In the latter setting, it was shown that migraine, although common and disabling, remains both an under-diagnosed and under-treated disease with the majority of sufferers to be under none of the available preventative treatment options at the time of that survey [13].

### Patients and methods

This population-based survey took place a few months after the outbreak of the COVID-19 pandemic and data collection occurred over a period of two months from June 1 to July 31 2020. No formal sample size calculation was done and the sampling process was based on random call to participants in replying to a specific migraine-focused questionnaire, including 151 questions in Greek language, which was distributed on a national level (all 13 regions) through the online research software SurveyMonkey. Migraine sufferers, regardless of being or not a member of the GSMHP, were encouraged to participate in the survey through the social media accounts (Facebook, Instagram and Twitter) of GSMHP and additionally, a dedicated invitation to participate was sent by e-mail to all members of the GSMHP. A reminder invitation was sent on June 30, 2020 to those initially invited to participate but have not responded.

The study comprised of two phases, i.e., the interview and then the online survey. Only adult patients, aged between 15–70 years old, with a definite diagnosis of migraine, were interviewed. As part of the random sampling process, participants were initially asked, during the interview, to provide their demographic data and residency to be able to capture the best possible representative sample of Greek migraine sufferers in terms of age, gender and geographical distribution and optimize sample’s representation.

Afterwards, participants were asked to reply to a screening question expressed as “do you have a definite diagnosis of migraine established by a physician or do you have clinical symptoms resembling migraine but not formally diagnosed by a physician?” and then completed key clinical questions to ascertain if the diagnostic criteria for a definite migraine diagnosis were fulfilled, according to the most recent iteration of the International Classification of Headache Disorders, 3rd edition (ICHD-3) [14].

**Fig. 1:**
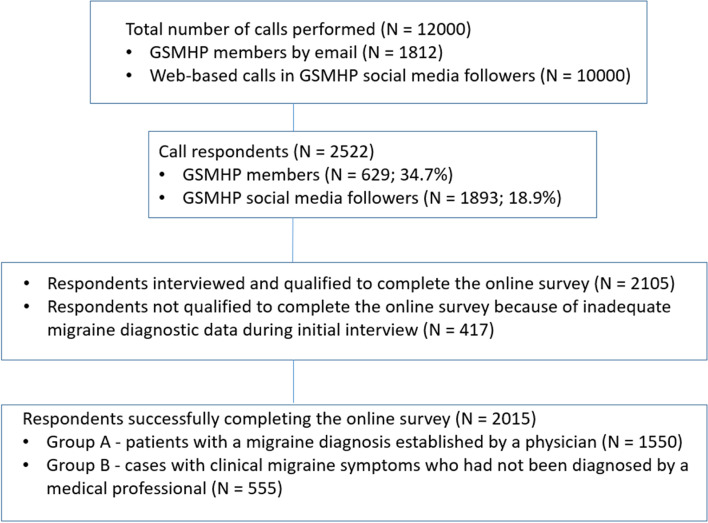
The flow diagram of participants

Participants providing during the interview inadequate migraine diagnostic data, according to both ICHD-3 symptom criteria and physician diagnostic criteria, were asked to stop filling-in the survey and withdraw. All others fulfilling the diagnostic criteria for migraine with/without aura were afterwards asked to continue by completing during the online survey phase all primary remaining questions in relation to the severity and effects of migraine on patients' QOL and daily living activities (working/societal), as well regarding the impact of the COVID-19 pandemic on the course of migraine. Finally, all patients who successfully completed the questionnaire were divided into two groups before extracting and analyzing data; to those with a diagnosis of migraine established by a physician (Group A), and to cases with clinical migraine symptoms who had not been diagnosed by a medical professional (Group B). The flow diagram of participants is presented in Fig. 1.

The research working group consisted of two members of GSMHP with qualification and experience in methodology, techniques and tools for conducting scientific research and four headache specialists who assessed and analyzed the extracted data of this survey.

Approval to conduct this survey was obtained from the ethics committee of Euromedica General Clinic, Thessaloniki, Greece. A mandatory consent question was included at the beginning of the Web-based survey and the participants were informed about the purpose of the survey, how the data will be used, that the data are anonymous and they had to read and to agree. The study was performed in accordance with the principles of the Declaration of Helsinki.

### Statistical analysis

Descriptive statistics presented categorical variables as observed counts and weighted percentages, and continuous variables as mean or median with the corresponding standard error or range, depending on their nature. For the statistical analysis, Mann–Whitney U test for two samples in non-parametric comparisons, Chi-square one-sample test and Chi-square with Yates corrected *P*-value in the comparison of proportions were used. All tests were performed using the SPSS for Windows (release 27.0; SPSS Inc., Chicago, IL). Significance was set at the *P* < 0.05 level.

## Results

### Demographical data

The study enrolled 2,105 patients who after completion of the first diagnostic part of the questionnaire fulfilled the diagnostic criteria for migraine, according to both the ICHD-3 symptom criteria and physician diagnostic criteria. The study sample consisted of 159 males (7.6%) and 1946 females (92.4%) with a mean age of 32.5 ± 14.3 (range: 18–60) years. Patients had failed a median number of 3 (range: 0 – 7) previous oral prophylactic medications. Comorbidities were common, being mostly clinically manifested as chronic pain syndromes (*N* = 286; 58.8%); hypo/hyperthyroidism (*N* = 211; 43.4%); irritable bowel syndrome (*N* = 126; 26.9%); depression (*N* = 99; 20.4%); diabetes and/or hypertension (*N* = 75; 15.4%) and asthma (*N* = 54; 11.1%).

Among the total of 2105 participants, 1550 of them had a diagnosis of migraine by a physician (Group A), and 555 cases were at baseline with clinical migraine symptoms who had not been diagnosed by a medical professional (Group B). The majority of our sample were women (92.45%; *n* = 1946). Of the patients, 1.05% (*n* = 22) were up to 18 years-old, 2.33% (*n* = 49) were 19–25 years-old, 10.45% (*n* = 220) were 26–34 years-old, 38.76% (*n* = 816) were 35–44 years-old, 42.23% (*n* = 889) were 45–59 years-old and 5.18% (*n* = 109) were 60 years and older. The duration of migraine (answers to the question: "How many years do you suffer from migraine?") was less than a year in 1.25% (*n* = 26), 1–5 years in 12.87% (*n* = 267), 6–10 years in 15.04% (*n* = 312) and more than 10 years in 70.84% (*n* = 1.470) of patients.

The age of the first experience of migraine symptoms (answers to the question "At what age did you first experience symptoms that turned out to be migraine symptoms?") was 0–12 years, 13–18 years, 19–25 years and over the 25 years for the 13.64%, 25.54%, 28.43% and 32.39% of the patients, respectively.

The subjective event that was deemed to associate with the onset of migraine (answers to the question "What important event do you think is related to the onset of your migraines?" was death of a loved one in 11.63% (*n* = 52), physical / psychological abuse in 10.74% (*n* = 48), the onset of menstruation in 8.28% (*n* = 37) and pregnancy or childbirth for 15.88% (*n* = 71) of patients. The rest of the patients (53.47%; *n* = 239) did not mention any specific event.

Family history of migraine (answers to the question "Is there a relative of yours who also suffers from migraines?" was reported by 1357 patients (65.56%), with 50.89% of this subgroup referring to their mother, a 19.38% to their father, a 21.38% to a sibling, a 11.24% to a child of theirs, a 21.38% to a grandparent and a 30.33% to an uncle / aunt / cousin (answer to the question "Which relative of yours suffers from migraine?").

### Diagnosis of migraine

A total of 75.15% answered positively to the question "Have you visited a doctor who has diagnosed that you are suffering from migraine?" qualifying as Group A-patients, with a 7.81% of them having been diagnosed by a physician, 87.35% by a neurologist, 1.68% by a general practitioner and a 1.55% by a doctor in a pain clinic (answer to the question "What is the specialty of the doctor that diagnosed your migraine?").

Similarly, the answer of Group-A patients to the question "What is the specialty of the doctor who has been consulting you for the last semester to a year)?" was "a neurologist" by a 88.72%; "physician" by a 4.31%; "doctor in a pain clinic" by a 1.49%; "general practitioner by the 1.16% and other specialty by a 4.31%. The answers to the question "How satisfied are you with the doctor who is following-up you lately and with the therapeutic options she/he suggested" were "not at all" by a 3.32%, "a little" by a 9.29%, "fair enough" by a 33.17%, "a lot" by a 26.87% and "very much" by a 27.36%.

### Characteristics and severity of migraine

**Fig. 2:**
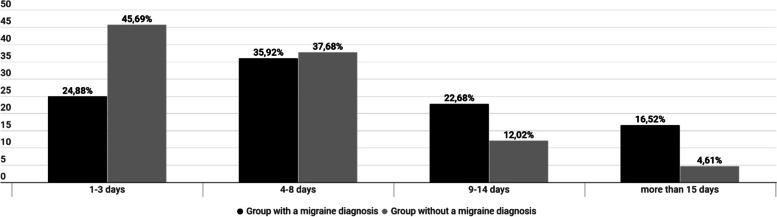
Answers to the question: *“How many days PER month (on average) do you experience migraine symptoms?*”. Differences between the patients with “migraine” diagnosis from medical professional and patients with no official diagnosis but clinical migraine symptoms

There were between groups differences in the frequency of migraine (answers to the question "How many days in a month (on average) do you experience migraine symptoms?" clarified to include all days with migraine symptoms, mild or severe, at a time not under prophylactic treatment (Fig. 2).

**Fig. 3:**
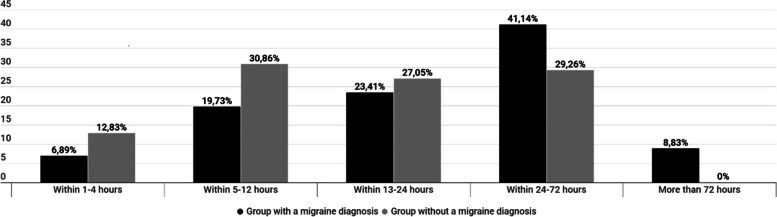
Answers to the question: “*How many hours does a migraine attack usually last if you do not take any painkiller or if it does not work?*". Differences between patients with “migraine” diagnosis from medical professional and patients with no official diagnosis but clinical migraine symptoms

Likewise, the answers of the two groups were different in the severity of migraine (answers to the question "How many hours does your migraine attack usually last if you do not take a painkiller or if you take one but would not work?", Fig. 3).

**Fig. 4:**
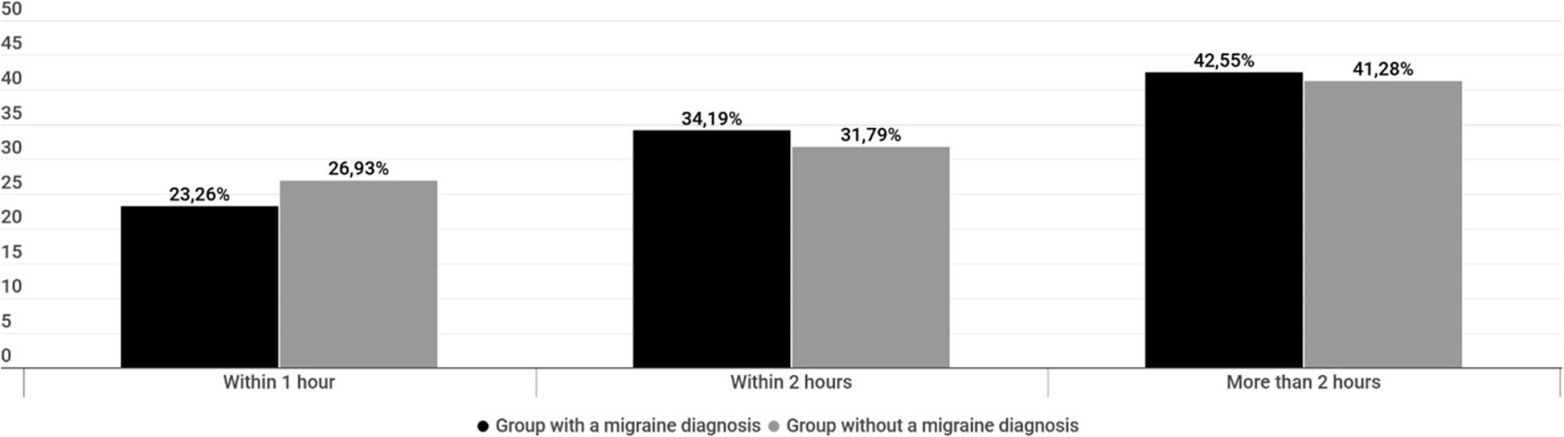
Answers to the question: “*How long does it take to get free from migraine after you receive a painkiller when it works?*”. Differences between patients with “migraine” diagnosis from medical professional and patients with no official diagnosis but clinical migraine symptoms

The answers of the two groups were very similar to the question "How long does it take to get free from migraine after you receive a painkiller when it works?" with the clarifications that pain and all other migraine symptoms have to be completely disappeared, and that the patients should answer this question only if they use painkillers for migraine relief (Fig. 4).

**Fig. 5:**
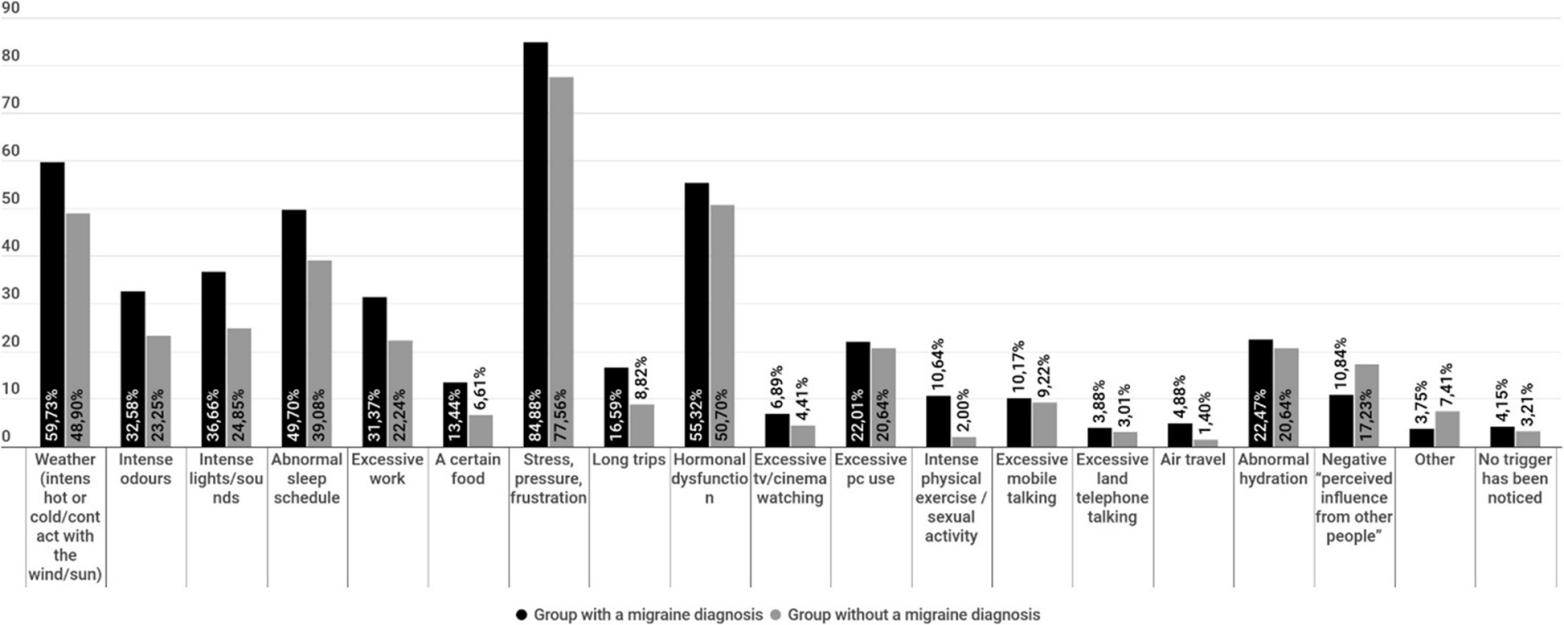
Triggers of migraine as they were reported by the participants. Differences between patients with “migraine” diagnosis from medical professional and patients with no official diagnosis but clinical migraine symptoms

The answers (multiple choice) of both groups to the question "Choose which factor(s) do you consider as the main trigger for your migraine" are shown on Fig. 5.

**Fig. 6:**
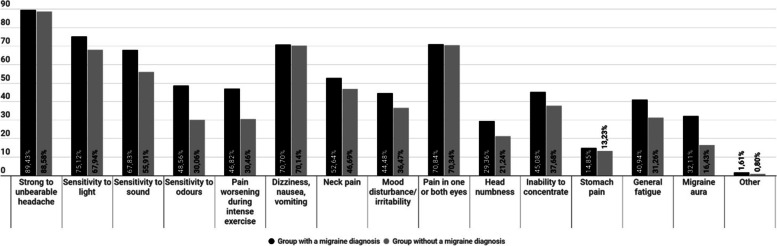
Symptoms during migraine attack as they were reported by the participants. Differences between patients with “migraine” diagnosis from medical professional and patients with no official diagnosis but clinical migraine symptoms

The answers (multiple choice) to the question "Which symptom(s) do you experience during a migraine attack?" are shown on Fig. 6.

### Social, work, financial and emotional consequences

According to the answers to the question (multiple choice) "Which of the following aspect(s) do you consider to be extremely negatively affected by your migraines?", emotional/ psychological impact was reported by a 72.20% of the patients, impact on family by a 68.29%, impact on their work by a 66.15%, impact on social domain (relationship with friends, leisure activities, etc.) by a 58.06%, financial impact by a 22.50% and impact on school or academic performance by a 13.18%. Respondents less or equal than 45 years old experienced more intense social, work and financial consequences, compared to those 46 or older (*P* = 0.01). The emotional/psychological impact remained unaffected by the age cut-off of 45 years old. All the above domains in relation to migraine burden were found to be more severely impacted in respondents with chronic vs episodic migraine (*P* < 0.001), as also in those highly educated with university degree and MSc/PhD (*P* = 0.03).

### Impact on work

The questions were addressed to employed patients. To the question "how many days in approximate per month are you absent from work due to migraine?", a 35.19% of the responders answered “1–2 days”, a 9.91% answered “3–5 days”, a 5.98% answered “more than 5 days”, and a 48.92% answered “none”.

To the question "How many days per month do you have a reduction in your performance at work due to migraine?", the 7.96% of the participants answered “none”, 38% “1–2 days”, 31% “3–5 days” and a 22.93% answered “more than 5 days”.

To the question "Which of the following has happened to you because of your migraine…?", to be answered only by patients who suffer from migraine for more than 15 days per month, the answer “I reduced my working hours” was given by a 14.77%, “I stopped working (on my choice)” by a 12.98% “I changed the subject of my work” by a 5.82%, “I took a long leave from my work” by a 3.58%, and “I lost my job” by a 2.91%. The majority (70.25%) answered none of the above.

A 11.22% of the participants answered “I reduced my working hours” to the question "Which of the following has happened to you because of your migraine…" (with the instruction to be answered only by patients who have less than 15 days of migraine per month), while a 5.40% answered “I changed the subject of my work”, a 4.74% “I stopped working”, a 1.33% “I lost my job”, a 1.08% “I took a long leave from my job” and a 80.38% “none of the above”. *Impact on family and social life*

To the question "Approximately how many days per month you are not able to meet your family obligations due to migraine", a 37.26% of the participants answered “1–2 days”, 25.48% “3–6 days”, 8.23% “7–10 days”, 5,65% “more than 10 days”, and a 23.39% “I don’t miss family obligations”.

To the question "Approximately how many days per month you are not able to meet your social obligations due to migraine", 42.80% of the participants answered “1–2 days”, 27.76% “3–6 days”, 9.54% “7–10 days”, 7.33% “more than 10 days”, and 12.56% “I do not miss family obligations due to migraine”.

### Emotional impact

The answer "…others do not understand that migraine is not just a simple headache" was given by the 70.44% to the question (multiple answers allowed) "I feel very often…". A 65.94% of the participants answered “…that I lose a part of my life in pain”, a 55.87% “…anxious for the next crisis, a 24.53% “…the need to hide my migraine from others”, a 24.04% “…that I disappoint those close to me”, a 21.01% “…depression and denial of life”, a 6.98% “… that friends / colleagues / family treat me differently because of my migraine”, a 7.15% “…that I lose friends because of my migraine”, a 3.25% “… suicidal thoughts” and a 7.80% gave various other answers.

**Fig. 7:**
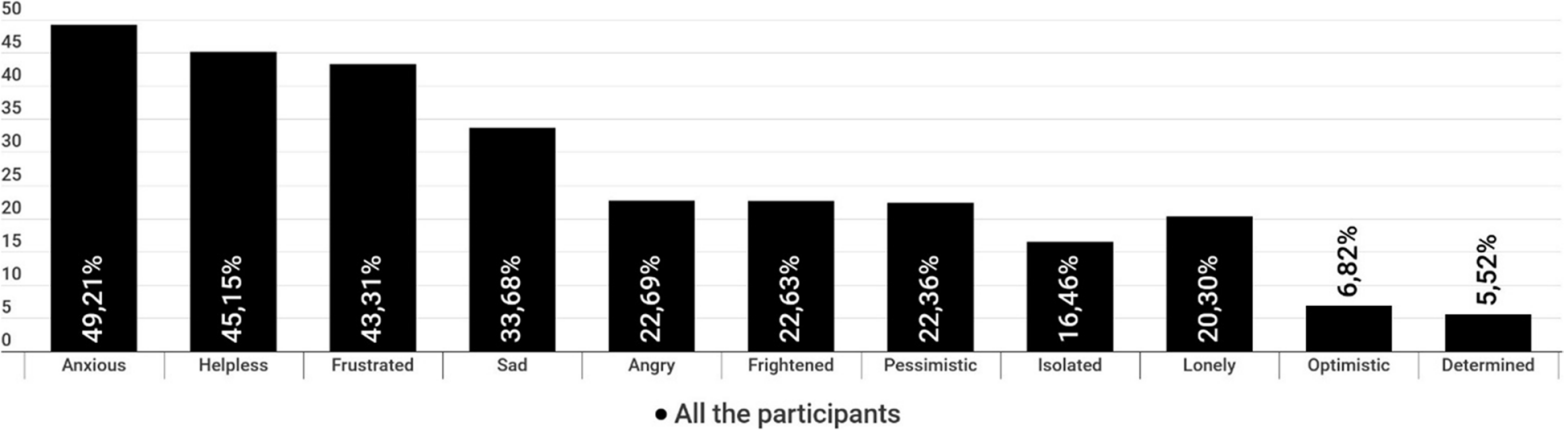
Answer to the question “choose up to 4 words from the following to describe how you feel about your life with migraine” (up to 4 answers per participant)

The multiple-choice question "Please, choose up to 4 words from the following to describe how you feel about your life with migraine" is shown in Fig. 7. Overall, negative emotional responses prevailed with “anxious” (49,2%), and “helpless” (45,15%) being the most common.

### Effect of coronavirus pandemic restrictive measures on the course of migraine

The question "Do you think that the COVID-19 pandemic restrictive measures had any impact on your migraine" was answered as “they had no effect on my migraine” by the 56.15%, “probably made it worse” by the 15.15%, “definitely made it worse” by the 10.65%, and on the contrary, a 14.38% answered “rather they improved it” and a 3.68% “they definitely improved it”. As can be seen in Table 1, respondents who said COVID-19 had no effect on migraine (56%), made it worse (26%) or claimed that migraine improved (18%), shared comparable characteristics in terms of monthly migraine frequency as also monthly days of acute headache medication use.

**Table 1:** Comparison of migraine incidence and acute medication monthly intake of respondents in relation to the effect of COVID-19 pandemic on their disease

Groups of respondents in relation to the effect of COVID-19 pandemic on migraine burden	No effect	Worst	Better	*P* value
Monthly migraine frequency				
(Mean ± SD)	7.2 ± 5.8	6.9 ± 8.5	7.1 ± 5.5	*P* = 0.52
Median	6	7	6	
Monthly days with acute medication use				
(Mean ± SD)	4.7 ± 3.7	5.1 ± 5.2	4.5 ± 3.4	*P* = 0.22
Median	4	5	4	

Moreover, a similar work, social impact and emotional impact was seen in the responders of each of the latter three categories.

The many-choices-allowing question "What exactly did cause the effect of the pandemic on your migraine" (given only to those who reported a negative effect) was answered as “the concerns and anxiety about the general situation of the country and the planet” by the 44.82%, “the health concerns for me or my relatives” by the 40.99%, “the too many hours of relaxation at home” by the 29.79%, “the cessation of working and related stress” by the 28.51%, “the concerns for my occupational future” by the 23.40%, “the reduced physical activity” by the 17.73%, “the long hours of sleep” by the 16.74%, “the use of mask” by the 14.75% and “the change in my eating habits” by the 13.62%.

Τhe answer to the question "Did anything change in relation to the treatment of your migraine throughout the period from the onset of the pandemic until now, due to restrictive measures (quarantine, curfews, etc.)?" was “I was not treated before the restrictions were imposed” by the 47.86% of the participants, “no, nothing changed in my treatment” by the 45.61%, “I did not have access to health services as before the pandemic” by the 3.13%, and “yes, my treatment changed after medical advice” by the 2.03% and “yes, my treatment changed on my own decision” by the 1.37%.

### Associations

When we examined potential associations of demographic and baseline variables, included in Table 2, with the clinical characteristics of our sample, we found gender effects with females to experience four times higher EM (odds ratio (OR) 3.8, *p* < 0.001) and five times higher CM (OR 4.9, *p* < 0.001) compared to males. Age effects were also noted in relation to the prevalence of EM which was higher in the age group 35–44 (OR 1.5, *p* = 0.01), while the prevalence of CM was higher in the 45–59 age group (OR 1.3, *p* = 0.03). We were not able to reveal any other significant differences in burden and clinical factors in relation to the other variables, including geographical distribution (*p* = 0.3) or even when we stratified our participants to those with basic school education (elementary – high school) compared to those with higher (university – PhD/MSc owners) education (*p* = 0.2).

**Table 2:** Describes in detail the baseline epidemiological and clinical characteristics of participants

Variable	Participants *n* = 2015 N %
Gender	
Females	1946 92.4
Males	159 7.6
Age ± SD (range)	32.5 ± 14.3 (18–60)
18yo and less	22 1.0
19-25yo	49 2.3
26-34yo	220 10.5
35-44yo	816 38.8
45-59yo	889 42.2
60yo and above	109 5.2
Level of education	
Elementary	27 1.3
Middle School	67 3.2
High School	829 39.4
University degree	860 40.8
Master/PhD owner	322 15.3
Regional geographical distribution	
Thrace	123 5.9
Macedonia	520 24.8
Epirus	75 3.6
Thesally	139 6.6
Ionian sea islands	40 1.9
Aegean sea islands	113 5.3
Peloponnese	165 7.8
Attica	727 34.5
Central Greece	82 3.9
Crete island	121 5.7
Years ± SD (range) with migraine	11.6 ± 6.5 (0.5–18)
Less than a year	26 1.2
1–5 years	267 12.9
6–10 years	312 15.1
More than 10 years	1470 70.8
Skipped answer	40
Monthly migraine days	
1–3 days/month	372 24.9
4–8 days/month	537 35.9
9–14 days/month	339 22.7
More than 15 days/month	247 16.5
Skipped answer	610

## Discussion

The "Greek Society of Migraine and Headache Patients" conducted during the COVID-19 pandemic, in 2020, its second online survey, coined, "Migraine in Greece 2020". The multifold purpose of this online, population-based study was to describe the characteristics of migraine patients in Greece, the severity, the effectiveness and satisfaction of the preventive and symptomatic treatments offered, the social, occupational, financial and emotional impact on everyday life of the patients, as well as the effects of the coronavirus pandemic on the course of the disease. It remained outside of our aims to provide epidemiological data and estimate the prevalence of migraine in Greece. As such, our data cannot be directly compared to the findings of the recently published study, by Constantinidis et al., on the prevalence and burden of disabling primary headaches in Greece, which was conducted using computer-assisted telephone interviews [12].

Nonetheless, our results confirm that the vast majority of patients with migraine are women. The corresponding percentage in the first similar survey (GSMHP conducted in 2018) was 93% [13] vs 92% in the current, while a survey from the European Migraine & Headache Alliance (EMHA) conducted in various European countries, recorded a percentage 89.98% of women [15]. Nonetheless, this overwhelming gender predominance of female over male respondents in the current and 2018 [13] settings, might also be explained by the assumption that female patients with migraine are likely more active in social media; thus, being more affable to actively participate in online, web-based surveys.

In addition, the age distribution of our sample allocated most of cases in the 36–59 age categories, similar to what was previously reported in 2018 [13]. In addition, the majority of patients (70.84%), suffer from migraine for more than 10 years, while the corresponding percentage in the 2018 survey was 65% [13]. In any case, it is a strong fact reflecting the perennial nature of migraine. Concerning the age at which patients are experiencing migraine for the first time, we found that 4 out of 10 patients experienced migraine attacks before they reached adulthood.

The genetic factor was strong, with 65.56% of patients answering that they had a relative also suffering from migraine, while that of the 2018 survey was 58.2% [13]. Concerning diagnosis of migraine, 3 out of 4 patients (75.15%) have been diagnosed by a doctor and the 87.35% of them was diagnosed especially by a neurologist. Satisfaction rates from the attending physician do not reveal any strong trend, but are recorded as positive.

A fact that is important to emphasize is that 50% of Group A patients had visited an emergency department due to a severe migraine attack, as well as 1 in 5 migraine cases from Group B. On the contrary, 15.25% of patients in Group A had to be hospitalized due to a migraine attack, while in Group B only 3.61% answered positively. Considering that prior studies have shown that if the population is screened for ICHD symptoms only about half of those with symptoms have ever been diagnosed with migraine by a physician [8, 9], another important of our findings to highlight is that a much lower percentage of interviewed respondents, i.e., 26%, met symptom criteria but were not diagnosed by a medical professional, whereas this effect is likely attributed to our sampling process, targeting advocacy group members.

Regarding the impact of migraine on patients’ quality of life, 7 out of 10 respondents stated that they experience emotional / psychological, family and/or occupational effects. In addition, 3 in 5 reported an impact on their social activities, 1 in 5 a financial impact and 1 in 10 an impact on their school or academic performance.

Regarding the effects on work due to disability experienced by an employed patient due to migraine, 1 in 3 stated that they lost 1–2 working days per month, while the corresponding percentage it our 2018 survey was 37.2% [13]. Concerning the days of reduced productivity at work, 38.03% of the participants experienced reduced productivity at work for 1–2 days per month. The corresponding percentage in our 2018 survey was 31.6% [13].

The impact on family and social life are more pronounced, presumably because the inability to participate in these obligations leads to milder consequences than absence from work. The financial cost is the reason for not having access to services or treatments for their migraine for 2 out of 5 of the participant patients.

On the emotional level, similarly with the 2018 survey, the overwhelming majority, 7 out of 10 participants, stated that they feel that others do not understand that migraine "is not a simple headache" and they also feel that they lose a part of their life in physical pain [13]. Six out of 10 people are anxious about their next attack, as well as 1 in 4 people feel the need to hide their migraine from others or that they disappoint their loved ones. Due to their illness, 1 in 5 people experienced depression and negativity for their life and a percentage of 3.25% reported suicidal tendencies.

Over the last three years there are several publications exploring the impact of restrictive measures with enforcement of lockdowns and other changes in social situations on migraine, during the COVID-19 pandemic, due to increases in anxiety, depression and sleep disorder rates as also because of patients’ difficulties to attend a regular follow-up [16–19].

Regarding the effect of the coronavirus pandemic and restrictive measures on the course of migraine in the current setting, more than 1 in 2 of our patients answered that they did not experience any effect on their migraine, while 1 in 4 reported a negative effect and 1 in 5 patients a positive one. This is an intriguing finding given that migraine has been very well associated with anxiety disorders while psychiatric comorbidities are significant migraine triggers [20] and as such one could expect that migraine indices would significantly differ before vs during the COVID-19 pandemic. Our findings do not support the latter view and we might thereby suggest that the coronavirus pandemic is a different kind of stressor on migraine sufferers compared to the stressors of everyday life. Moreover, data disclosed in our 2018 pre-COVID-19 survey [13] on migraine severity, triggers, symptoms, impact on work, impact on family and social life and emotional impact, remain comparable to current data obtained during the pandemic in relation to its effect upon the burden of migraine. Nonetheless, we should mention that he sampling process we applied in the 2018 and current survey to target advocacy group members and not patients followed in our headache outpatients’ clinics, might also have influenced this specific finding, while a more focused look into domains captured in the assessment, i.e., work, social, emotional impact, would have potentially uncover unrecognized burden.

In a Hawaiian study on the early impact of the COVID-19 pandemic in migraine patients on an outpatient basis, 33.1% of 118 patients reported increased migraine symptoms severity or frequency during the restrictions (16.1% of 118 patients used more acute migraine treatment), 63.6% had no change in their migraine profile) [21]. These findings are comparable with those of our study (25.8% and 56.15%, respectively). Regarding any changes in the treatment of the participants’ migraine due to the pandemic and the restrictive measures, in our study 1 in 2 stated that they were not treated before the imposition of restrictive measures and 1 in 2 stated that nothing changed in their treatment. A very small percentage, 3.13% of patients, reported that they did not have access to health services, such as botulinum toxin injections for chronic migraine or other treatments that require a doctor or clinic. In contrast, another web-based study on 1018 migraine patients during the same time period reported an increase in migraine severity in 64.1% and an increase in frequency in 59.6% of the participants [19], while 61.5% reported having no communication with their treating physician for the whole duration of the pandemic. Possible causes in patients with increased migraine were different than in our study: 4.9% were afraid to lose their job (23.4% in our study), dietary habits changes were reported as a reason for worsening in 58.3% (13.6%), sleep disturbances in 78.1% (16.7%), and reduction of physical activity in almost 80% (17.7%) [19]. In our study the negative effect on migraine had more to do with general health, social habits and financial concerns. Nevertheless, sampling differences, country differences in healthcare, timing of data collection are to be acknowledged as possible reasons for differences in findings between different studies.

Our survey possesses, several limitations, including the use of subjective rather than objective collection of data using an internet-based survey, the inclusion of cases with a tentative diagnosis of migraine at baseline (group B), the cross-sectional study design and the focus of our survey to participants with a long history of episodic or chronic migraine having access to social media. Moreover, we should acknowledge the limitations associated with the potential sampling bias from targeting individuals with diagnosed migraine in the recruiting process and that advocacy group members were also targeted. Finally, we should mention that the questions used in the assessment have face validity but were not validated or subjected to any form of psychometric process.

Nonetheless, our survey showed that migraine has a significant impact on a patient's quality of life. The severity of migraine depends on the frequency of days per month that the patient experiences with migraine, the intensity of pain, the specific symptoms and the response to treatment. When the frequency of days with migraine increases, the intensity can lead the patient to the emergency department or hospitalization. On the other hand, when the effect of the acute treatment of migraine decreases, the severity of the disease and the social professional, financial and emotional impact increase. Migraine was probably not significantly affected in general from the current coronavirus pandemic situation regardless of its psychological burden. Further larger longitudinal population-based studies are warranted to further explore the demographics and clinical phenotypic characteristics of migraine patients in Greece as well as in a multinational level.

## Data Availability

The corresponding author has full control of all primary data and agrees to allow the journal to review our data upon reasonable request.
